# Factors that influence the selection of conservative management for end-stage renal disease – a systematic review

**DOI:** 10.1093/ckj/sfad269

**Published:** 2023-10-17

**Authors:** Pavithra Sakthivel, Alyaa Mostafa, Olalekan Lee Aiyegbusi

**Affiliations:** College of Medical and Dental Sciences, University of Birmingham, Birmingham, UK; College of Medical and Dental Sciences, University of Birmingham, Birmingham, UK; Centre for Patient Reported Outcomes Research, University of Birmingham, Edgbaston, Birmingham, UK; Institute of Applied Health Research, University of Birmingham, Birmingham, UK; National Institute for Health Research (NIHR) Birmingham Biomedical Research Centre (BRC), University of Birmingham, Birmingham, UK; NIHR Applied Research Collaboration (ARC) West Midlands, Birmingham, UK; Birmingham Health Partners Centre for Regulatory Science and Innovation, University of Birmingham, Birmingham, UK; NIHR Blood and Transplant Research Unit (BTRU) in Precision Therapeutics, University of Birmingham, Birmingham, UK

**Keywords:** conservative care, conservative management, end-stage renal disease, ESRD, non-dialysis

## Abstract

**Background:**

Most patients with end-stage renal disease (ESRD) are managed with dialysis and less commonly kidney transplantation. However, not all are suitable for or desire either of these renal replacement therapies. Conservative management (CM) is an option. However, the selection of CM is often not easy for patients and clinicians. The aim of this systematic review is to identify the key factors that influence the selection of CM for ESRD.

**Methods:**

Medline, Embase, PsychINFO, and CINAHL Plus were systematically searched from inception to 10 September 2021. Titles/abstracts and full texts were independently screened by two reviewers. Reference lists of included articles were searched. An update search via PubMed was conducted on 10 August 2023. A narrative synthesis of review findings was conducted.

**Results:**

At the end of the screening process, 15 qualitative and 8 survey articles were selected. Reference checking yielded no additional relevant studies. Main themes were: (i) Patient-specific factors; (ii) Clinician-specific factors; (iii) Organisational factors; and (iv) National and international factors. Patient-specific factors were awareness and perceptions of CM and dialysis, beliefs about survival, preferred treatment outcomes and influence of family/caregivers and clinicians. Clinician-specific factors included perceptions of CM as ‘non-intervention’, perceptions of clinician role in the decision-making process, and confidence and ability to initiate sensitive treatment discussions. Relationships with and involvement of other healthcare professionals, time constraints, and limited clinical guidance were also important factors.

**Conclusions:**

An improvement in the provision of education regarding CM for patients, caregivers, and clinicians is essential. Robust studies are required to generate crucial evidence for the development of stronger recommendations and guidance for clinicians.

KEY LEARNING POINTS **What was known:**

- Dialysis is generally regarded as a treatment modality that could extend the lives of patients with end-stage renal disease (ESRD).
- However, it may not confer any survival benefit on some geriatric patients and potentially worsen their quality of life.
- Understanding the key factors that may influence decisions to choose conservative management (CM) could improve treatment decision-making.

**This study adds:**

- Patients generally have limited awareness and information about CM as a treatment option for ESRD.
- Some healthcare professionals may be reluctant to offer CM to patients as they perceive it as ‘non-intervention’.
- There is a strong financial incentive for nephrologists to promote dialysis at US treatment centres.

**Potential impact:**

- Our review highlights the need to educate patients and develop appropriate guidance and training on CM for healthcare professionals.
- Healthcare professionals may utilize our findings to inform discussions with patients about treatment options and promote shared decision-making.

## INTRODUCTION

Most patients with end-stage renal disease (ESRD) are managed by dialysis and, less commonly, kidney transplantation. Although dialysis is generally regarded as a treatment modality that could extend patients’ lives, not all patients are suitable, and the burden of treatment may be substantial and outweigh its benefits [1, 2]. Furthermore, there is evidence that it may not confer any survival benefit on some geriatric patients and potentially worsen their quality of life and functional status [1, 3]. For these patients, conservative management (CM) is an option. The aim of CM is to manage symptoms and delay disease progression without the use of dialysis. It involves active medical and lifestyle interventions such as medications to mitigate the complications of renal failure and dietary modifications to maintain patients’ quality of life.

However, the selection of CM is often not easy or straightforward for patients, their caregivers and healthcare professionals (HCPs). While there are guidelines on the management of ESRD, detailed recommendations pertaining to CM are limited due to a lack of strong evidence [4–6]. Terminological inconsistencies and the interchangeable use of related but distinct terms such as ‘palliative care’ and ‘supportive care’, has led to misunderstandings about what constitutes (non-dialytic) CM [4]. There is a need to understand how the decision to undergo CM is made and what factors influence this process. Comprehensive insights into perspectives of individuals across different contexts can be gained by synthesizing multiple qualitative and quantitative studies [7, 8]. The aim of this systematic review is to explore and summarize the key factors that influence the selection of CM as a treatment modality for ESRD.

## MATERIALS AND METHODS

The Enhancing Transparency of Reporting the Synthesis of Qualitative Research (ENTREQ) framework was used for the reporting of this review [7].

### Search strategy and article selection

Four databases – namely, Medline, Embase, PsychINFO (all accessed via OVID) and CINAHL Plus (via EBSCO*host*) – were systematically searched from their inception (1946, 1974, 1967, and 1937 respectively) to 10 September 2021, using a search strategy initially developed for Medline and subsequently adapted for the other databases (Table S1, see online supplementary material).

All titles/abstracts were independently screened by two reviewers (P.S./A.M. and O.L.A.) and those that did not meet the inclusion criteria were excluded. Full texts of potentially relevant studies were retrieved and assessed independently by the reviewers for eligibility. Discrepancies during title/abstract screening or full text evaluation were resolved through discussions among the reviewers. An update search via PubMed was conducted on 10 August 2023.

Qualitative and survey-based studies exploring patient, caregiver, and clinician experiences of the decision to commence CM for ESRD were eligible. Eligible studies were conducted in adult patients aged 18 years old and over. Articles focusing only on renal transplantation and/or dialysis without discussing CM, abstracts, and systematic reviews were excluded from the review. Only English language papers were included. Reference lists of included articles were searched.

### Data extraction

Data regarding study design and results were independently extracted by P.S. and A.M. for each eligible study. Data extraction was also conducted by both reviewers and cross-checked by O.L.A. for accuracy. A pre-designed form was used to extract data on study characteristics and findings.

### Critical appraisal

The methodologic quality of each of the eligible studies was independently assessed by P.S. and A.M. and cross-checked by O.L.A. Relevant items from the Consolidated criteria for Reporting Qualitative health research (COREQ) framework [9] were used to critically appraise the comprehensiveness of the reporting of each primary study as previously employed by Tong *et al.* [10]. These items pertained to the research team, study methods, setting, analysis, and interpretations. Discrepancies were resolved through discussion.

### Data analyses and synthesis

Due to the heterogeneity of the included studies, a narrative synthesis of the review findings was conducted based on the methods described by Popay *et al.* [11]. This involved: (i) a descriptive summary of the information extracted on study characteristics and critical appraisal; (ii) the exploration of associations and pooling of reported findings within individual studies, as well as across studies; and (iii) discussion of the findings and provision of recommendations for future research and clinical practice.

## RESULTS

### Characteristics of included studies

**Figure 1:**
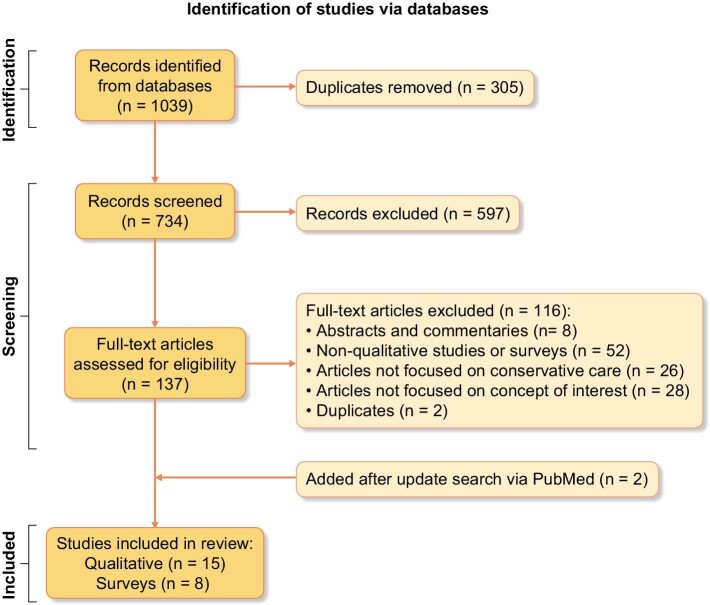
PRISMA flow diagram.

Our search retrieved 1039 entries (Embase–540, Medline–266, CINAHL Plus–159, PsycINFO–74). After removing duplicates, 734 titles/abstracts were independently screened. Of these, full texts for 137 were screened and 15 qualitative and 6 survey articles selected for this review. The update search via PubMed retrieved 188 entries. Following full text screening, two survey articles [15, 16] met our inclusion criteria, bringing the total number of included studies to 23. Reference checking yielded no additional relevant studies. The process and reasons for exclusions are presented in the PRISMA flow diagram (Fig. 1). Table 1 presents the characteristics of included articles while Table S1 (see online supplementary material) provides details of their appraisal.

**Table 1:** Characteristics: of included studies.

Study	Country	Sample size	No. females (%)	Mean age in years (standard deviation)	Data collection method	Data analysis	Key findings
Chanouzas *et al.* [25]	UK	118	N/A	HD: 68.1 (14.2), PD: 55.4 (13.7), CM: 84.1 (5.5)	Survey	Statistical analysis	Key factors differentiating patients who chose CM from those who chose HD/PD are age and co-morbidity index. Patients found written and oral information provided by HCPs valuable. It was also important for them to know whether they would be able to cope with their current lifestyle if they chose CM.
De Jong *et al.* [15]	Europe	7820	3465 (44.3)	59.2 (14.0)	Survey	Statistical analysis	25% of the patients had received no information on the different modalities, and only 23% received information >12months before KRT initiation. Patients were not informed about home haemodialysis (HHD) (42%) and conservative management (33%).
Eneanya *et al.* [12]	USA	31 (10 patients, 5 carers, 8 nephrologists, 8 primary care physicians)	Clinicians: 5 (21) Patients: 4 (40) Carers: 5 (100)	Clinicians: 44.8 (12) Patients: 73 (5.9) Carers: 62.8 (7.7)	Semi-structured telephone interviews	Narrative analysis	Major themes were dialysis discussions and decision-making. Nephrologists were more comfortable with management of disease whereas PCPs felt that they should be responsible for advocating for patients. Patients and caregivers were uncertain about some aspects of their care.
Grubbs *et al.* [3]	USA and UK	59 nephrologists	14 (23.4)	≤45 years: 34 46–65 years: 20 ≥66 years: 5	Semi-structured interviews	Narrative and thematic analysis	Nephrologists acknowledged that research does not show a significant improvement in life expectancy among elderly patients who opt for dialysis. They also recognized infrastructure, guidelines and training as important factors influencing the likelihood of introducing conservative care pathways to patients. Variable access to these resources meant patients did not receive standardized care from clinicians. Clinicians were hesitant to make absolute statements in the absence of reliable tools to predict prognosis. However, this did not mean that all treatment options were discussed impartially and presented information to patients in an optimistic and hopeful light.
Hamroun *et al.* [16]	France	136 nephrologists from 38 nephrology facilities, 1204 patients	81 (59.5) nephrologists	Nephrologists: 43	Survey	Statistical analysis	All participating facilities reported they were routinely able to offer conservative care, but only 37% had written protocols and only 5% had a person or team primarily responsible for it. Overall, 6% of patients were estimated to use conservative care. Among nephrologists, although 82% reported they were fairly or extremely comfortable discussing conservative care, only 28% usually or always offered this option for older (*>*75 years) patients approaching kidney failure.
Han *et al.* [24]	Singapore	23 (16 patients, 7 carers)	Patients: 9 (56.3) Carers: 5 (71.4)	Patients: 75	Semi-structured interviews	Inductive and deductive thematic analysis	Decision-making factors reported by patients: caregiving and financial burden, alternative medicine, lack of autonomy in daily life, symptom, and disease progression. Some reported that they were heavily persuaded by family members and/or doctors to undergo dialysis. Others stated they felt they had no choice, i.e. decision was taken by clinician.
Johnston *et al.* [23]	UK	9 patients	5 (55.5)	86	Clinic consultations with renal nurse specialist.	Constant comparison	Patients opting for CM seemed content with their decisions. They felt equipped to take these decisions after receiving explanations and discussions with clinical nurse specialists. Reasons for opting for CM included age, constant travel to hospital (for dialysis), burden to others and generally feeling that they will be well enough without dialysis.
Karlin *et al.* [18]	USA	21 (15 patients of which 7 not on dialysis and 8 on dialysis; 6 family members)	No dialysis: 4 (57.1) Dialysis: 2 (25) Family members: 6 (100)	No dialysis: 73.6 Dialysis: 71.4 Family: N/A	Semi-structured interviews	Systematic narrative, thematic analyses and comparative analyses	There is a common theme amongst the participants that the only option besides carrying out dialysis is death (this also seems to be their perception of conservative management) although they have also been made aware that dialysis could be of little benefit in certain situations. Other patients have mentioned that they had no choice in deciding their treatment. Overall, there is a lack of understanding regarding the treatment options available for kidney failure amongst both patients and family members.
Ladin *et al.* [27]	USA	35 nephrologists	7 (20)	N/A	Semi-structured interviews	Narrative and thematic analysis	Clinicians were expected to provide patients an active intervention that would better patients’ health and conservative management was perceived as a failure or giving up. This invoked feelings of fear and discomfort as clinicians were not willing to upset or disappoint patients. Varying competence in communication skills meant not all clinicians discussed controversial topics like conservative management with patients. Nephrologists expressed the requirement for multiple healthcare services to work together and the inaccessibility of such services as a barrier to recommending CM.
Morton *et al.* [21]	Australia	105	46 (43.8)	N/A	Survey	Statistical analysis	Key factors that influenced decision making were survival benefit, flexibility of dialysis, hospital visits and time spent, as well as restrictions on daily activities.
Noble *et al.* [26]	UK	27 healthcare professionals (12 nephrologists, 15 specialist nurses)	22 (81.5)	30–39 years: 6 40–49 years: 14 50–59 years: 3	Semi-structured interviews	Thematic analysis	Nephrologists appreciated the complexity of deciding on a treatment and encouraged patients to deliberate options in their own time. They recognized that the unpredictable course of the condition made it difficult for patients to understand the severity of their CKD. Clinicians recognized that influence of family members and friends in decision making and experienced moral dilemmas especially when patients and the inability to accurately predict disease progression added to uncertainty about which treatment would be more beneficial.
Oestreich *et al.* [17]	USA	20 (14 patients, 6 care-givers)	Patients: 4 (28.6) Caregiver: 4 (66.7)	Patients: 79 (7) Caregivers: (2)	Cognitive interviewing	Inductive thematic analysis	Patients were more willing to accept CM when presented as an active, holistic treatment option that will allow maintain quality of life and allow control over one's health. Some factors that made CM a less desirable option included: uncertainty over prognosis and perception as a passive ‘last resort’ pathway. Patients wished to have information about disease progression and life expectancy. They also mentioned personal values and information from close persons and clinicians also plays a crucial role in decision making.
Russell *et al.* [1]	USA	54 nephrologists and fellows	28 (51.8)	Nephrologists: 47 (11) Fellows: 33 (4)	Interviews and focus groups	Constant comparative method and thematic analysis	Internal conflicts between patients and their carers and the limited time available to comprehensively discuss conservative care are the two main factors named by nephrologists and fellows as barriers to discussing non-dialysis care.
Saeed *et al.* [20]	USA	13 patients	4 (30.7)	81.8 (7.3)	Information obtained from electronic medical records	Independent chart reviews	Main reasons for choosing CM were limited life expectancy regardless of dialysis initiation, expectations of poor quality of life on dialysis, dignity on end of life and peace of mind, experiences of witnessing others suffer on dialysis.
Scott *et al.* [29]	UK	60 (22 nephrologists, 25 nurses, 1 palliative care consultant, 12 allied healthcare professionals)	47 (78.3)	Median: 49 (range: 28–67)	Semi-structured face-to-face or telephone interviews	Secondary thematic analysis	HCPs considered various factors when recommending conservative management for patients such as the balance between risks and potential discomfort against the benefits of treatment, the predicted changes to quality of life and availability to clinical and personal resources.
Seah *et al.* [19]	Singapore	9 patients	4 (44.5)	Median: 81 (range: 61–84)	Semi-structured interviews	Iterative thematic analysis	Patients expressed full ownership in decision making and were hesitant to discuss their decision with HCPs due to fear of being pressurized to choose RRT. They also considered the money and time requirements and support needed from family/friends a burden. Patients were elderly and believed they had led a fulfilling life, making an invasive treatment option unnecessary.
Selman *et al.* [14]	UK	20 patients	9 (45)	Median: 82 (range: 69–95)	Semi-structured interviews	Inductive thematic analysis	Patients choose CM as they wished to maintain their quality of life and found dialysis time and resource intensive. They also have varying perspectives about their own involvement and the involvement of HCPs in decision making.
Tam-Tham *et al.* [31]	Canada	409	157 (38.4)	N/A	Survey	Statistical analysis	There is a need to enhance access to support for maintain the patient in the home setting to avoid transitions of care, which could be accomplished, in part, through advance care planning. There is also a need to provide educational support for patients, family, and PCPs. Furthermore, there is a need to increase telephone access by primary care physicians for direct and timely communication with nephrologists and experts in CM.
Tonkin-Crine *et al.* [13]	UK	42 patients	14 (33.4)	82	Semi-structured interviews	Inductive thematic analysis	Patients expressed the desire to maintain their quality of life and they found the logistical struggles of dialysis too great to balance its benefits. They had varying knowledge about their own condition and available treatment options.
Tsai *et al.* [32]	Taiwan	598	N/A	N/A	Survey	Statistical analysis	There was adequate understanding and interest amongst participants regarding CM. There were mismatches between interest, willingness, and knowledge of CM relative to the provision of CM in practice. The decision to provide CM can be partially influenced by the healthcare reimbursement infrastructure, such that higher compensation for dialysis treatments can form a constraint for nephrologists and nurses to act differently from their preference towards CM for some patients.
Van de Luijtgaarden *et al.* [30]	Belgium, Croatia, Germany, Finland, Greece, UK, Italy, Spain, Macedonia, Netherlands, Romania	433	151 (35)	N/A	Online survey	Statistical analysis	Nephrologists from non-profit and low RRT incidence care settings were more likely to consider patient preferences for treatment modality highly during decision making. They were also more likely to consider factors such as patient co-morbidities and discuss CM with patients and their families.
Verberne *et al.* [22]	The Netherlands	99 (dialysis: 75, CM: 24)	Dialysis: 21 (28) CM: 11 (46)	Dialysis: 79.8 (4.3) CM: 84.2 (4.9)	Survey	Statistical analysis	Despite the overall high satisfaction, older patients with advanced CKD had contrasting experiencing for both dialysis and CM. Decision-making was much more positive in the CM group. Many patients found it difficult to discuss CM with their relatives or explain their decisions to them.
Wachterman *et al.* [28]	USA	20 nephrologists	5 (25)	50	Semi-structured interview	Grounded and iterative thematic analysis	Dialysis was easier to discuss with patients. Others were concerned that patients are more likely to regret their decision of choosing CM over dialysis. Some nephrologists resort to shifting decision-making to patients, initiating time-limited trials of dialysis and/or convincing patients to choose dialysis.

CKD: chronic kidney disease; CM: conservative management; ESRD: end-stage renal disease; HCP: healthcare professional; HD: haemodialysis; RRT: renal replacement therapy; PCP: primary care physician; PD: peritoneal dialysis.

### Themes

**Figure 2:**
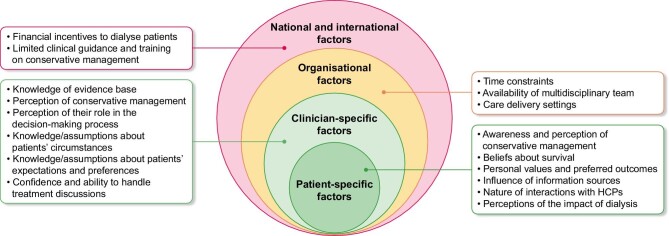
Themes and subthemes identified.

Four main themes were identified, namely: (i) patient-specific factors; (ii) clinician-specific factors; (iii) organisational factors; and (iv) national and international factors. The themes and sub-themes are presented below. Figure 2 illustrates the themes and subthemes. Illustrative quotes are provided in Table S2 (see online supplementary material).

### Patient-specific factors

#### Awareness of conservative management

Patients’ understanding of progression of kidney disease and ESRD was variable (Table 2). When discussing renal replacement therapy (RRT) with patients, clinicians rarely discussed CM and so their knowledge of CM was generally limited [12–16]. Patients were generally keen to know more about CM and some who heard about CM for the first time were surprised that their current care regimen (dialysis) had been a tacit decision without a balanced discussion of all treatment options [17]. Patients managed at centres with established CM pathways generally understood their illness trajectory better, received more information about CM, and often felt it was a viable option [13].

**Table 2:** Other indirect factors.

Understanding of chronic kidney disease (CKD) and ESRD
Patients’ understanding of CKD and progression to ESRD was variable. However, at some point during their illness trajectory, most patients with CKD generally received information about dialysis modality, setting, access placement, and frequency of treatments from their clinicians [12, 17, 19]. Patients considered access to verbal/written information about treatment modalities particularly important [25]. Some patients believed they had no CKD symptoms attributing all their symptoms to aging or other co-morbidities [13]. These patients often considered themselves well and tended to refuse dialysis when it was suggested by their clinicians and so might have found CM appealing without fully grasping its implications. For some patients who initially chose conservation management, worsening symptoms or a limited understanding of disease progression later led to revisions of treatment decisions from CM to dialysis [13, 24]. Misinformation was an issue in some instances, where conflicting or imprecise information was provided by HCPs.
**Perceptions of the impact of dialysis** *Impact on daily activities and lifestyle* The ability to continue performing normal daily activities was highly valued by patients and they wished to choose a treatment option that allowed this [21, 25]. Patients also wished to maintain autonomy and independence over their own life [12, 17, 20, 24]. Some patients had not previously considered the possibility that the burden of dialysis might outweigh the benefits [17]. Awareness of this possibility was validating for those who were already sceptical of dialysis based on their perceptions of its impact on their daily lives [17]. *Practicalities of dialysis* For some patients their reluctance to choose dialysis was due to the practicalities. Issues such as the inconvenience of frequent travel or the lack of transportation to dialysis units and lack of space for setting up bulky dialysis equipment were highlighted [13, 14, 19, 21, 23, 25]. *Time and financial implications* The potentially time-consuming nature of dialysis was an issue for some patients [15, 17, 20, 23]. Patients, particularly those who opt for CM, may perceive the time spent on dialysis as a waste of their time [13, 19]. However, patients already on dialysis might accept the time commitment and come to regard it as inconsequential [13]. Many patients believed the financial, time and physical cost of dialysis outweighs the benefits [19, 21]. Travelling for haemodialysis two or three times per week and spending hours each time was off-putting for most [19, 24]. Caregivers on the other hand did not consider these issues as enough reasons to reject dialysis and were often willing to contribute or meet the financial costs of dialysis [24]. *Burden on family/caregivers* Several patients on dialysis required the assistance of family members to undergo treatment. They required informal care and travel assistance to attend hospital appointments [13, 19]. Therefore, patients often considered dialysis burdensome not only to them but also to their family members/caregivers. For these reasons, some patients opted for CM [13, 23, 24]. This way they felt they were able to shield loved ones from the trauma and suffering of witnessing them undergoing dialysis [20]. As with financial costs of dialysis, family members did not consider this a sufficient reason to decline dialysis [24].

#### Perceptions of conservative management

There were variations in patients’ perceptions of CM possibly linked to their knowledge of what it entails [17]. While some considered CM as an alternative to dialysis, others did not consider them as mutually exclusive options [17]. Some patients found the concept of CM difficult to grasp, equating it to ‘doing nothing’ [18]. Some considered CM as supplementary or temporary/preparatory strategy that might be changed if/when their condition deteriorates [13, 17]. These patients felt there should be flexibility to start dialysis if their personal goals and circumstances changed over time [17].

Patients were sensitive to how CM was framed during discussions with clinicians [17]. However, CM was not always presented positively [13, 18]. Framing CM as a passive approach, where a decline in health was expected, led to a negative perception among patients [17]. However, describing it as an active approach to delay disease progression and enable patients to live well without dialysis, led to positive perceptions of CM. These positive views were associated with feelings of hope, and relief from previously assumed inevitability of dialysis [17]. At some centres, patients felt clinicians actively tried to discourage them from choosing CM by associating it with death while presenting dialysis as the option that could prolong their lives [13, 18, 19]. Patients’ views of the impacts of dialysis could also influence their decision to choose CM. Table 2 presents patients’ views of dialysis.

#### Beliefs about survival

While patients generally wanted information about their illness trajectory and survival on CM, several were wary of definitive statements based on personal experiences where their renal function had made small improvements contrary to the predictions by nephrologists [17]. These patients believed life expectancy is impossible to predict with any certainty and appreciated discussion with clinicians who acknowledged this. For some patients, the uncertainty itself was a source of hope that they may have a favorable outcome making CM more appealing [17]. Patients were more likely to opt for CM when informed that dialysis may not extend their lives [17].

#### Personal values and preferred outcomes

The ability to maintain their current lifestyle and quality of life were important considerations for patients during the decision-making process [13, 14, 17, 18, 20–22]. A longer life span was generally not a priority for elderly and frail patients. They preferred CM and the maintenance of their quality of life to undergoing ‘aggressive’ dialysis to prolong their lives [13, 15, 20, 22, 23]. They mostly expressed a sense of contentment, ‘life completion’ and lesser anxiety about death accepting it as the eventual outcome of their condition or advanced age [14, 17, 19, 20, 22, 23]. They preferred what they considered a ‘dignified life closure’ [20].

#### Interactions with clinicians

Patients highlighted the importance of being able to discuss treatment options freely with clinicians [14]. They generally preferred clinicians to be more proactive in providing information about all options and answering their concerns and queries [14, 20]. It was critical that information was provided by clinicians in an honest, unambiguous, easy to understand yet sensitive and personal manner [14].

#### Influence of family members and clinicians

Most patients felt the decision to choose CM was ultimately theirs to make [19, 20, 22–24]. However, the views of family members and clinician recommendations often influenced patients’ treatment decisions [14, 17, 25]. These individuals sometimes had powerful influences on some patients, making them feel they had no choice but to accept dialysis [24, 26]. A survey of patients who underwent a decision-making process for dialysis or CM found that more patients who selected dialysis felt forced to make the decision, mostly due to their deteriorating health or by their nephrologist [31% (dialysis) vs. 5% (CM), *P* = 0.01] [22].

For patients who declined dialysis, the unpleasant experiences of friends and family members on dialysis often carried more weight than clinician recommendations (which were mostly pro dialysis) [19, 20, 23, 24]. Some of these patients were reluctant to inform their clinicians of the decision due to fears of being pressurized to change their minds [14, 19].

### Clinician-specific factors

#### Knowledge of the evidence on conservative management

Nephrologists readily acknowledged that research demonstrates similar life-expectancy for some patient subgroups such as elderly patients including those with dementia or ischemic heart disease irrespective of treatment choice. This made them more likely to recommend CM to such patients [3]. There were indications that with growing evidence to support CM, attitudes are changing, especially among recently qualified nephrologists [3].

#### Perceptions of conservative management

Clinicians generally perceived dialysis as an ‘active’ treatment modality that could prolong the life of patients [27]. They reported feelings of failure or helplessness when patients could not be offered dialysis [3, 27]. The majority considered CM as the last line of treatment options, with some regarding it as ‘no treatment’ and a failure of medicine [27].

Several clinicians highlighted the difficulty predicting the potential outcomes of dialysis or CM. They recollected instances of patients who have done well on dialysis despite their age or frailty [3, 26, 28]. Therefore, they were uncomfortable discussing these uncertainties and were more inclined to recommend a trial of dialysis in the first instance to all patients even if they thought CM was a better option [1, 26, 28, 29].

#### Perception of their role in the decision-making process

Clinicians’ perception of their role in the decision-making process varied. Some took on active roles which ranged from guiding patients to near-coercion while others preferred to leave the decision almost entirely to patients and their families [26, 28]. A survey of nephrologists found that although 81% of the respondents reported discussing CM with all their patients with ESRD, they only recommended it to 10% of patients [30].

A US study reported that nephrologists and primary care physicians (PCPs) believed that as nephrologists were more knowledgeable about patient suitability for dialysis, they should initiate discussions about RRT and manage all aspects of care specific to kidney disease [12]. Some of the PCPs also felt that even though they might be of the opinion that dialysis was unsuitable for some patients, these decisions should be made by a nephrologist. Others thought it should be a shared decision in conjunction with a nephrologist. PCPs generally expected to play a supportive role by providing continuity of care [12, 31], ensuring that patients understood the impacts of their chosen treatment modality and helping them navigate the healthcare system [12]. It was important to PCPs that they can contact nephrologists and access conservative clinic services when required [31].

#### Knowledge/assumptions about patients’ circumstances

Clinicians often considered individual patient circumstances. They were more inclined to suggest CM to older patients, and in the presence of frailty and/or life-limiting co-morbidities [12, 26, 29, 30]. Some considered the mental as well as the physical health of their patients and suggested CM to patients they believed were emotionally capable of handling the choice [29]. Nephrologists appreciated the influence of patients’ social support structures, education, family and friends and financial ability on their willingness to choose CM [26, 29]. They also acknowledged that well-meaning families pressurising clinicians and patients into dialysis is a barrier to discussing CM [1, 26, 27].

#### Knowledge/assumptions about patients’ expectations and preferences

Many clinicians were hesitant to recommend CM due to their assumptions of patient expectations of care. They felt bound by patients’ expectations of receiving an active intervention to help them ‘get better’ [1, 27]. Some clinicians believed they could predict patients’ values and preferences and considered it their duty to guide them to choose a treatment in line with their best interests [3, 26, 27]. This meant that clinicians did not always present all treatment options in a neutral manner [3, 13, 27]. Clinicians also made assumptions about the level of information patients would understand or like to receive [26].

#### Confidence and ability to handle treatment discussions

Clinicians highlighted difficulty dealing with patients’ emotional responses to information about treatment options as an issue. This meant that they sometimes withheld information that patients might find upsetting which might lead to a negative perception of a preferred option [1, 26, 27]. Some clinicians lacked confidence and admitted being hesitant about engaging patients and family/caregivers in sensitive discussions around CM and death [1, 3, 12]. Variations in approach and clinicians’ expertise/skill in dealing with sensitive and difficult conversations often led to non-uniform care provision [3]. Some American nephrologists highlighted the issue of historical mistrust of healthcare systems among African-American patients as a potential barrier to the acceptance of clinician recommendations to forego dialysis. This might also make clinicians hesitant and less likely to recommend CM to these patients [3, 27].

### Organisational factors

#### Time constraints

A major barrier to discussing CM was time constraint due to clinician workload. Poor patient awareness of CM often meant multiple consultations were required to help them understand it as an option. In some cases, the time required to build rapport with patients meant that sufficient time to fully discuss CM was not available [1, 27]. Initiating dialysis was considered the easiest option, especially for hospitalized patients [3].

#### Interactions with and support from other HCPs

UK and US nephrologists considered their interactions with other HCPs as an important factor. Some were concerned that recommending CM might be queried or perceived negatively by their peers [3] and in the case of trainees, their seniors [1]. The involvement of other HCPs who may or may not have expertise in renal care could also be an issue [31]. There may be a lack of support from other HCPs who often expect dialysis and may offer to patients before consulting nephrologists [1, 3, 27].

#### Care setting

Availability of appropriate services and HCPs for the delivery of CM is essential [3, 27, 29, 31]. Nephrologists felt they required the support of multidisciplinary teams to offer CM [16, 27]. As most renal centres have well established pathways for haemodialysis, this is often the convenient or only option [29, 30].

### National and international factors

#### Limited clinical guidance and training on conservative management

Nephrologists from England and the United States cited limited clinical guidance and training on CM as some of the reasons they recommend dialysis to most patients by default [3, 27]. Lack of or limited training meant significant variations in attitudes and approaches to CM [27]. In a survey, Canadian PCPs highlighted the need for access to educational resources to enhance the support they provide to patients and family members during the decision-making process [31]. A survey conducted in Taiwan indicated that HCPs, particularly nephrologists, dialysis nurses, and palliative care specialists were keen to learn more about CM and the selection of appropriate patients [32]. Following completion of the training courses, 87.1% of the participants expressed their willingness to provide CM in future [32].

#### Financial incentives to dialyse patients

American nephrologists pointed out that there was a strong financial incentive to dialyse patients. Keeping patients alive for as long as possible was financially rewarding for dialysis centres [1, 3, 27]. The issue was compounded by a lack of reimbursement for time required for in-depth discussions about CM. Therefore, to maximize their income, several American nephrologists prioritized consultations with as many patients as possible in short time periods [3].

A European survey found that nephrologists based at not-for-profit centres significantly rated patient preference as an important factor in decisions about CM higher than those based at for-profit centres (*P* = 0.03) [30]. UK nephrologists stated that the funding mechanisms of the National Health Service (central for dialysis and local for non-dialytic management) could lead to fewer resources for adequate non-dialytic programmes in rural areas [3].

## DISCUSSION

This study explores and summarizes the factors that influence the selection of CM for patients with ESRD. Patients’ views of CM may be influenced directly or indirectly by the kind and level of information they receive from clinicians. Some patients indicated that they would prefer doctors to play a more proactive role in the decision-making process and expressed frustrations with reticent doctors. For some patients, knowledge of the experiences of individuals already on dialysis and their perceptions of its impact may have a greater influence on their own treatment decisions than discussions with clinicians. Our finding that patients often receive little or no information about conservative management from HCPs during discussions about treatment options was in line with the findings reported by previous studies [33, 34]. However, perennial organisational issues, stemming from limited resources such as time constraints for clinical appointments could limit the amount of information about CM that clinicians are able to provide patients and caregivers.

We found that older patients were more inclined to opt out of dialysis, preferring CM that prioritizes and preserves their quality of life while not necessarily prolonging their lives. This was confirmed by the review conducted by Shi *et al.* [35]. Nevertheless, in settings with limited or inadequately coordinated multidisciplinary care, and no established care pathways for CM, dialysis in the absence of serious contraindications might be the easiest or only option.

The limited guidance on CM might explain why some clinicians are reluctant to recommend it from the outset. A recent systematic review concluded, based on a limited number of studies, that patients who receive CM may have improved mental health-related quality-of-life compared to those dialysed [36]. However, there is a need for stronger evidence from well-designed primary studies (including quantitative and qualitative cohort studies) which will reassure patients and clinicians, facilitate shared decision making and the development of firmer clinical guidance.

Our review highlights the need for appropriate training and resources on CM for HCPs involved in the decision-making process for patients with ESRD. Clarifications are required on what constitutes palliative, supportive or CM. Furthermore, misconceptions that CM is non-treatment or should only be considered as a last resort should be dispelled. The term ‘comprehensive conservative care’ proposed following a consensus process at the 2013 KDIGO Controversies Conference on Supportive Care in CKD and its definition needs to be fully understood and adopted [4, 37]. Comprehensive conservative care is an active approach that requires effective coordination of multidisciplinary involvement [4, 37].

The majority of the studies included in this review were conducted in western countries, therefore the findings may not be entirely applicable to other settings where cultural and socio-economic issues might exert greater influences [38]. There is a possibility that our search did not retrieve a few relevant articles, or we excluded some due to terminological inconsistencies. Despite these limitations, our review provides a detailed overview of the key factors that may influence the selection of comprehensive conservative care. Healthcare professionals may find these insights valuable when discussing treatment options with patients and utilise the knowledge to facilitate shared decision-making and the provision of patient-centred care.

## Supplementary Material

sfad269_Supplemental_File

## Data Availability

The data underlying this article are available in the article and in its online supplementary material.
