# Supplementary material for: Factors that influence the selection of conservative management for end-stage renal disease – a systematic review
Source: Clin Kidney J. 2023 Oct 17;17(1):sfad269. doi: 10.1093/ckj/sfad269 (PMC10768754; doi:10.1093/ckj/sfad269)
Supplement: sfad269_Supplemental_File [file sfad269_supplemental_file.docx]

**Table of Contents**

Table S1 – Critical appraisal of qualitative studies

Table S2 - Illustrative Quotations by Theme and sub-themes

| **Table S1. Appraisal of the comprehensiveness of reporting in included qualitative studies** | | |
| --- | --- | --- |
| **Item** | **Studies reporting each item** | **Number of studies (%)** |
| **Personal characteristics** |  |  |
| Interviewer/facilitator identified | Saeed et al.,^1^ Eneanya et al.,^2^ Han et al.,^3^ Johnston et al.,^4^ Karlin et al.,^5^ Oestreich et al.,^6^ Seah et al.,^7^ Selman et al.,^8^ Tonkin-Crine et al.,^9^ Grubbs et al.,^10^ Ladin et al.,^11^ Noble et al.,^12^ Wachterman et al.,^13^ St Clair Russell et al.^14^ | 13 (86.7) |
| Occupation of interview/facilitator | Saeed et al.,^1^ Eneanya et al.,^2^ Johnston et al.,^4^ Oestreich et al.,^6^ Scott et al.,^15^ Seah et al.,^7^ Selman et al.,^8^ Tonkin-Crine et al.,^9^ Grubbs et al.,^10^ Ladin et al.,^11^ Noble et al.,^12^ Wachterman et al.^13^ | 12 (80.0) |
| Experience/training in qualitative research | Saeed et al.,^1^ Eneanya et al.,^2^ Han et al.,^3^ Johnston et al.,^4^ Karlin et al.,^5^ Scott et al.,^15^ Selman et al.,^8^ Tonkin-Crine et al.,^9^ Ladin et al.,^11^ Noble et al.,^12^ Wachterman et al.,^13^ St Clair Russell et al.^14^ | 12 (80.0) |
|  |  |  |
| **Relationship with participants** |  |  |
| Relationship established prior to start of study | Saeed et al.,^1^ Eneanya et al.,^2^ Han et al.,^3^ Johnston et al.,^4^ Karlin et al.,^5^ Scott et al.,^15^ Selman et al.,^8^ Tonkin-Crine et al.,^9^ Noble et al.,^12^ Wachterman et al.^13^ | 11 (73.3) |
|  |  |  |
| **Participant selection** |  |  |
| Selection strategy (e.g., snowball, purposive, convenience, comprehensive) | Saeed et al.,^1^ Eneanya et al.,^2^ Han et al.,^3^ Johnston et al.,^4^ Karlin et al.,^5^ Oestreich et al.,^6^ Scott et al.,^15^ Seah et al.,^7^ Selman et al.,^8^ Tonkin-Crine et al.,^9^ Grubbs et al.,^10^ Ladin et al.,^11^ Noble et al.,^12^ Wachterman et al.,^13^ St Clair Russell et al.^14^ | 15 (100.0) |
| Method of approach or recruitment | Saeed et al.,^1^ Eneanya et al.,^2^ Han et al.,^3^ Johnston et al.,^4^ Karlin et al.,^5^ Oestreich et al.,^6^ Seah et al.,^7^ Selman et al.,^8^ Tonkin-Crine et al.,^9^ Noble et al.,^12^ Wachterman et al.,^13^ St Clair Russell et al.^14^ | 12 (80.0) |
| Sample size | Saeed et al.,^1^ Eneanya et al.,^2^ Han et al.,^3^ Johnston et al.,^4^ Karlin et al.,^5^ Oestreich et al.,^6^ Scott et al.,^15^ Seah et al.,^7^ Selman et al.,^8^ Tonkin-Crine et al.,^9^ Grubbs et al.,^10^ Ladin et al.,^11^ Noble et al.,^12^ Wachterman et al.,^13^ St Clair Russell et al.^14^ | 15 (100.0) |
| Number and/or reasons for nonparticipation | Eneanya et al.,^2^ Han et al.,^3^ Johnston et al.,^4^ Oestreich et al.,^6^ Seah et al.,^7^ Selman et al.,^8^ Tonkin-Crine et al.,^9^ Noble et al.^12^ | 9 (60.0) |
|  |  |  |
| **Setting** |  |  |
| Venue of data collection | Saeed et al.,^1^ Eneanya et al.,^2^ Han et al.,^3^ Johnston et al.,^4^ Karlin et al.,^5^ Seah et al.,^7^ Selman et al.,^8^ Tonkin-Crine et al.,^9^ Grubbs et al.,^10^ Noble et al.,^12^ Wachterman et al.^13^ | 12 (80.0) |
| Presence of nonparticipants (e.g., clinical staff) | Han et al.,^3^ Selman et al.^8^ | 2 (13.3) |
| Description of sample | Saeed et al.,^1^ Eneanya et al.,^2^ Han et al.,^3^ Johnston et al.,^4^ Karlin et al.,^5^ Oestreich et al.,^6^ Scott et al.,^15^ Seah et al.,^7^ Tonkin-Crine et al.,^9^ Grubbs et al.,^10^ Noble et al.,^12^ Wachterman et al.,^13^ St Clair Russell et al.^14^ | 13 (86.7) |
|  |  |  |
| **Data Collection** |  |  |
| Use of questions, prompts, or topic guide | Eneanya et al.,^2^ Han et al.,^3^ Johnston et al.,^4^ Karlin et al.,^5^ Oestreich et al.,^6^ Scott et al.,^15^ Seah et al.,^7^ Selman et al.,^8^ Tonkin-Crine et al.,^9^ Grubbs et al.,^10^ Ladin et al.,^11^ Noble et al.,^12^ Wachterman et al.,^13^ St Clair Russell et al.^14^ | 14 (93.3) |
| Repeat interviews/observations | Saeed et al.^1^ | 1 (6.7) |
| Audio/visual recording | Eneanya et al.,^2^ Han et al.,^3^ Karlin et al.,^5^ Oestreich et al.,^6^ Scott et al.,^15^ Seah et al.,^7^ Selman et al.,^8^ Tonkin-Crine et al.,^9^ Grubbs et al.,^10^ Ladin et al.,^11^ Noble et al.,^12^ Wachterman et al.,^13^ St Clair Russell et al.^14^ | 13 (86.7) |
| Field notes | Saeed et al.,^1^ Han et al.,^3^ Seah et al.,^7^ Selman et al.,^8^ Tonkin-Crine et al.,^9^ Wachterman et al.^13^ | 6 (40.0) |
| Duration of data collection | Saeed et al.,^1^ Eneanya et al.,^2^ Han et al.,^3^ Johnston et al.,^4^ Karlin et al.,^5^ Oestreich et al.,^6^ Grubbs et al.,^10^ Ladin et al.,^11^ Noble et al.,^12^ Wachterman et al.,^13^ St Clair Russell et al.^14^ | 11 (73.3) |
| Data (or theoretical) saturation | Eneanya et al.,^2^ Han et al.,^3^ Johnston et al.,^4^ Karlin et al.,^5^ Oestreich et al.,^6^ Scott et al.,^15^ Tonkin-Crine et al.,^9^ Grubbs et al.,^10^ Ladin et al.,^11^ Wachterman et al.,^13^ St Clair Russell et al.^14^ | 10 (66.7) |
|  |  |  |
| **Data analysis** |  |  |
| Researcher/expert triangulation (multiple researchers involved in coding and analysis) | Saeed et al.,^1^ Eneanya et al.,^2^ Han et al.,^3^ Karlin et al.,^5^ Oestreich et al.,^6^ Seah et al.,^7^ Selman et al.,^8^ Tonkin-Crine et al.,^9^ Grubbs et al.,^10^ Ladin et al.,^11^ Noble et al.,^12^ Wachterman et al.,^13^ St Clair Russell et al.^14^ | 12 (80.0) |
| Derivation of themes or findings (e.g., inductive, constant comparison) | Saeed et al.,^1^ Eneanya et al.,^2^ Han et al.,^3^ Johnston et al.,^4^ Karlin et al.,^5^ Oestreich et al.,^6^ Seah et al.,^7^ Selman et al.,^8^ Tonkin-Crine et al.,^9^ Grubbs et al.,^10^ Ladin et al.,^11^ Wachterman et al.,^13^ St Clair Russell et al.^14^ | 13 (86.7) |
| Use of software (e.g., NVivo) | Eneanya et al.,^2^ Han et al.,^3^ Karlin et al.,^5^ Oestreich et al.,^6^ Scott et al.,^15^ Selman et al.,^8^ Tonkin-Crine et al.,^9^ Grubbs et al.,^10^ Ladin et al.,^11^ Noble et al.,^12^ Wachterman et al.^13^ | 11 (73.3) |
| Member checking (participant feedback on findings) | Wachterman et al.^13^ | 1 (6.7) |
|  |  |  |
| **Reporting of quotes/raw data** |  |  |
| Participant quotations or raw data provided | Saeed et al.,^1^ Eneanya et al.,^2^ Han et al.,^3^ Johnston et al.,^4^ Karlin et al.,^5^ Oestreich et al.,^6^ Scott et al.,^15^ Seah et al.,^7^ Selman et al.,^8^ Tonkin-Crine et al.,^9^ Grubbs et al.,^10^ Ladin et al.,^11^ Noble et al.,^12^ Wachterman et al.,^13^ St Clair Russell et al.^14^ | 15 (100.0) |

| **Table S2.** **Illustrative Quotations by Theme and sub-themes** | |
| --- | --- |
| **Themes and Sub-themes** | **Illustrative Quotations** |
| **Theme 1: Patient-specific factors** | |
| Understanding of CKD and ESRD | “Staff wanted to put me on dialysis, nearly five months ago, but I didn’t want to go on dialysis. Everything is all right, you know, I don’t have to go on dialysis.”^9^  “The nurse said ‘we’ve given you a score of 6 [GFR].’ I thought—6/10, that’s not bad. Then I thought, 6 out of how many? She said ‘6/100 that’s how poorly you are’ and that brought me down to earth. [Later in interview] It went from 6 to 5 and the doctor said, ‘don’t worry, it’s alright,’ she says, ‘I’ve got a patient on 4, been on 4 for years and she’s still alive, don’t worry.’”^9^  “I don’t think it’s an agonising death. they said ‘you could suddenly start to feel very ill. and then ultimately probably go into a coma and just disappear.’ Which doesn’t sound pleasant but it’s not that bad to worry about.”^9^ |
| Awareness of conservative management | “I’ve never really thought about it. Well, all I know is that I have kidney failure, and if you had no kidneys, what are you going to do? You’re just going to die. So, the only alternative is dialysis”^2^  “It was presumed that dialysis would work for me…I can’t remember [staff] ever suggesting or saying that there is a third option – of not having dialysis.”^9^  “[The staff] said ‘it’s up to you, you’ve got the choice. You can have dialysis or you can have the other thing. if you want not to have dialysis it’s your choice but you’ve got to realize that it is going to kill you.but if you’re on dialysis you could last for ten, fifteen, twenty years’.”^9^ |
| Perceptions of conservative management | “We’re talking about not care as oppose[d] to care through dialysis and I have a tendency to be black and white.”^6^  “So you’re just waiting to die? It doesn’t sit well with me.”^6^  “It’s a little bit melancholy… what it’s saying is there’s really nothing that can be done..that you’re on your own.”(Oestreich et al., 2021)  “What it’s for, to treat the symptoms and preventing or managing medical problems that might result from damaged kidney function, protecting remaining kidney function. Help a person live well without dialysis … That statement makes me feel positive, kind of anticipating maybe a good outcome … I felt good about it … It gives you hope.”^6^  “I think treatment is diet, exercise, continued support from other people. I mean those are all things that are treatments … That makes the difference between life and death as far as I’m concerned … I will do whatever it takes for me to continue.”^6^  “It indicates to me that I have to make a choice between conservative care and dialysis, that I cannot have both. I think they work together, I would think. I don’t know why they’re separating it out.”^6^  “These things are something with conservative care, you do all of this before you need the dialysis.”^6^ |
| Beliefs about survival | “I decided that I didn’t want dialysis. I’m told that’s not terribly unusual and I was told that if you say yes to dialysis, you don’t necessarily live any longer anyway.”^6^  “So many things can go wrong…from a philosophical standpoint, it’s just as well that life expectancy is difficult to predict.”^6^  “It’s on the informative side, it’s telling you that even though you can’t predict what’s going to happen, the outcome will still be the outcome…it’s pretty matter of fact.”^6^ |
| Personal values and preferred outcomes | “If I was younger…in my forties or fifties, I will opt for dialysis…but not now at this age, where I don’t have a care in this world.”^7^  “I’m too old now, that dialysis thing is more for younger people…Not for me”^4^  “The clinic people have been very, very helpful and answered questions that I wanted to know about but aged 93…if I was 39 … I would think about it twice but at the moment I think we will let nature take its course and I will be quite happy with that. Is that alright?”^4^  “I am already 60 plus years old, my children are big…There is no need for me to consider so much.”^7^  “His goals at this age are to maintain dignity and avoid the invasion of his body with dialysis and other aggressive medical procedures. He made a choice to pursue CM for his and his family’s peace of mind, and to preserve the dignity of his body and his soul.”^1^  “Extending this type of life is no good. You bear for the pain, you got to take medicine everyday, then go for dialysis for some days; rest of the days are gone. You are tired, you cannot go anywhere”^3^ |
| Interactions with clinicians | “They’ve been very good, all the way, just ask anything and they tell you. There’s never been any problems like that, or not finding out what you wanted or anything.”^8^  “If it’s a direct order from the doctor, then I would force him to have it. If his doctor told me, ‘Look, your husband needs it now’ [then I would say] ‘It’s the doctor’s order, not mine, and you need it!”^5^  “there is nothing to discuss with the doctor. The doctor is wary and persuaded me to accept dialysis… all they do is encourage me to go on dialysis and tell me the benefits of dialysis.”^7^  “You know, an elderly, frail patient, you’re not going to overload them with a lot of scary complications and side-effects because you might potentially upset them…So, again, there’s a wee bit of bias in patient selection as to how much negative information you want to give a patient.”^12^ |
| Influence of family members and clinicians | “I've seen many people here in wheelchairs who keep drooling and need people to feed them; to be honest it's better to die than to lead such a life.”^3^  “I have seen my friends go through dialysis and the shows on television. The people on dialysis look so weak and helpless.”^7^  “This is because I have seen others on dialysis, and it looks like they are suffering. I’ve seen friends around the neighbourhood who went for dialysis, they were rather active prior to dialysis… but after going through dialysis”^7^  “Some of the doctors that I talk to…say, well if it had been them they’d have probably made the same decision, so that reinforces the decision I made was right, and I made it on my own.”^8^  “Unfortunately, he was beginning to get uremic and beginning to lose capacity and it all completely unraveled. I feel at that point that the family had made a decision for that patient, that the patient hadn’t necessarily made themselves but the patient had lost capacity at this stage to make his own decision.”^12^  “Some families just demand ‘I want my dad to dialyse’, they don’t give their relative a chance to decide … It’s very hard because the family can be very, very strong … ‘No, I decide, you know – I am the head of the family and I decide’”^12^ |
| Perceptions of the impact of dialysis | *Impact on daily activities and lifestyle*  “I’d never given any thought to the possibility that dialysis may cause more discomfort and one would feel like it’s not worth it.”^6^  “Let me lead as close to type of lifestyle as I would have if I didn’t have health issues going on – just allowing me to do what I can as long as I can is a form of support for me.”^6^  “I don’t want to waste a week of my life all the time when I can be at home, enjoying myself. I mean, to me, I’m going to lose my life if I’m going to have to be on dialysis.”^9^  “I’m satisfied with the way things are going. I’ve avoided dialysis. Dialysis doesn’t really do anything, it just compensates. Dialysis doesn’t really improve anything.”^6^  *Practicalities of dialysis*  “I can’t drive and I live out of town so it’s relying on hospital transport and I mean you could be waiting hours.. I just couldn’t cope with it.”^9^  “She showed me the bottle of stuff and I thought…where am I going to put all that?...I’ll have that in my bungalow?”^8^  *Time and financial implications*  “Rich people have the luxury to spend their money on dialysis in order to extend their lives by 1-2 years, but for poor people like us, the sooner we pass on the faster we will be released from our burdens.”^7^  “It’s very troublesome. I need to go to the dialysis centre 2-3 times a week, it’s a waste of time, you want me to sit there for 2 hours. It’s enough to scare me half to death.”^7^  “I’m 81 so it don’t matter to me, I thought four hours out of your life twice a week, what difference does it make? I would only be sat watching the television anyway.”^9^  *Burden on family/caregivers*  “Even if your son is capable, he has to take care of himself and his own family. Does he still have to take care of you? Can you bear to ask him to take care of you? If he starts taking care of you, he cannot stop taking care of you; but if he continues taking care of you, it's a problem too.”^3^  “He initially refused to undergo dialysis and delayed it for a long time because he didn't want to pose a burden on our children”^3^  “I made the final decision because I'm the person in trouble, they support”^3^  “Money is a small issue, because there are financial aids, the main problem is burden on family…I thought that my wife would have a hard time and a lot of stress, she needs to take care of the whole family…”^7^ |
| **Theme 2: Clinician-specific factors** | |
| Knowledge of the evidence on conservative management | “There’s literature out there that says dialysis in those elderly folks with poor functional status doesn’t really improve survival. That’s what I tell them.”^10^  “The younger faculty here seem to be a little bit more in tune with the idea that not everybody needs dialysis which obviously as you know becoming more and more evidence-based potentially in literature while others [older faculty] are just saying, ‘Well, they need dialysis. We’re going to dialyze them.’”[American nephrologist]^10^ |
| Perception of conservative management | “The thought that conservative care is no treatment is a (stopping) point for conservative care. It sort of feels like...feels like giving up*.*” Having “nothing to offer”^11^  “Some of the patients who don’t have capacity perhaps, who we have best interest meetings and then the decision is for the patient actually to have dialysis, and it…has worked really well” (Social Worker, Unit 3)^15^ |
| Perception of their role in the decision-making process | “We say it’s always about patient choice, but there’s no doubt that there’s a large medical steer in that. You know, if you show someone all the options, a lot of the time, they say, you know, ‘What do you think?’ and obviously, you can guide people down a path because you think it’s the right thing for them.”^12^  “No. I just usually use my own clinical knowledge, depending on their comorbidities. Sometimes it’s clearly obvious what’s best for them and you don’t need a decision aid by any stretch of the imagination [laughing]. Sometimes, the nurses will be part of the decision aid … But no, we don’t have any formal policies, as it were.”^12^  ‘‘It’s [the patient’s] decision. They cannot expect me to force a treatment on their family member. That’s what I find sometimes challenging.’’ (Nephrologist)^14^  “I have had patients who have refused to engage in dialysis planning, and they’re clearly not wanting conservative management. And I have been very naughty and said ‘Look, if we don’t move forward with this dialysis planning, the alternative is you don’t get dialysis and I’m sending you to somebody to talk about what it means not to have dialysis’, and I’ve done that, and then they come back and they say, ‘Right, okay, I’ll have dialysis now … You may say that’s very naughty, but it’s helpful for the patient’”^12^  “He was unconscious, very ill and in his eighties. You’d say this man’s too sick to do anything, but we talked to his family and they said, no, they wanted to give him the chance at dialysis, and we did. He lived for at least another three years at home with his family and was eternally grateful – every time I came in, he was ‘Thank you’, oh … and it was like, well, I just don’t know. We don’t know what’s going to happen in any individual patient, so you cannot be too sort of, judgmental on what to do.”^12^ |
| Knowledge/assumptions about patients’ circumstances | “For patients who are over 75, who have got lots of illnesses…dialysis isn’t guaranteed to make them live longer or feel better…those patients who have got a number of comorbidities, so patients who have got heart failure, COPD, dementia…conservative management is very, very appropriate.”^15^  “There are often very significant vascular [paths], amputations, heart attacks, strokes – and they sometimes have a significant element of cognitive decline – and I know they may well get worse on dialysis.”^15^  “It is more difficult to keep people well on dialysis treatment if they have poor social support it is also more difficult to maintain good quality of life for them with conservative management.” (Consultant Nephrologist, Unit 5)^15^  “I’ve had one or two cases where they [carers] don’t want them [patients] at home, they would rather pack them off for haemodialysis… It just gives them the opportunity to have a bit of a rest” (Staff Nurse, Unit 6)^15^ |
| Knowledge/assumptions about patients’ expectations and preferences | “It was hard…realizing that he [was] at the end of his - at the end of his rope - at the end of his line. He still wanted to try dialysis, even though in my heart of hearts I kind of knew this was gonna be the outcome.”^13^  “I tell [patients] you could always try dialysis and see if it’s something that works for [you], makes [you] feel better. Even if they don’t love the dialysis, are they enjoying their other days when they’re not on dialysis?...And then if after, let’s say, a month or two they say, ‘You know, Doc, this is just not for me,’ I tell them that I will a hundred percent support them in their decision to…stop the dialysis and just try to deal with the symptoms and comfort care.”^13^  “He[patient]was frightened by the prospect of a decline in his functional status due to dialysis or even just a reduction in a chance at improved functionality”^1^  “If the family says, ‘We want everything done’ and automatically that means that you have to do dialysis. For me it’s that we want everything done but for somebody…who is declining. We wouldn’t do brain surgery if we knew it wasn’t going to save them.” [American nephrologist]^10^  “You know, an elderly, frail patient, you’re not going to overload them with a lot of scary complications and side effects because you might potentially upset them … So, again, there’s a wee bit of bias in patient selection as to how much negative information you want to give a patient.”^12^ |
| Confidence and ability to handle treatment discussions | “We do discuss non dialytic care to CKD5; however I think that there’s huge variability in who really lays that out for them. Some don’t seem to either believe in it, or they don’t feel comfortable in approaching that topic and opening up that level of discussion, which is again, you know just a lack of uniformity in the we approach some…patients.”^10^  “I think even just saying, ‘well, you may become nauseated or you may get increasing swelling or things like that,’ is very different than saying, ‘and eventually you will die from your kidney disease.’ Just saying those words is a lot harder.” (Nephrologist)^14^  “We [renal care provider] don’t feel... comfortable to go and approach the patient and ask because you always think it’s best to come up from a doctor and usually its discussed with the family sitting around and discussing with their you know their diagnosis and their other medical issues and explaining more properly than for you think you probably don’t have that knowledge to go and talk to someone …… always think it’s best to come out from a doctor”^2^  “There are people for instance that practice medicine in a hospital that has been in existence from the 1800s and up until the late 1960s or 1970s, people of African-American heritage were not very trusting for a good reason…. It’s not that way anymore, but there are people still alive today that remember the 60s and find it very difficult to give their trust in a physician that comes out of that system.” [American nephrologist]^10^ |
| **Theme 3: Organisational factors** | |
| Time constraints | “And you can just imagine that if it was a conversation that was – they knew was going to be difficult and time consuming, they wanted to give that patient the time, that it might just be easier to delay that because they’ve got so many other things to do.”^2^ |
| Interactions with and support from other healthcare professionals (HCPs) | “…If a cardiac surgeon does an open heart [surgery] in an 85-year-old and the patient develops renal failure tomorrow how can I come and say, ‘I don’t want to dialyze this patient because she’s 85,’ or something like that. So, what am I supposed to do at that time?” [American nephrologist]^10^  “I would tell my colleagues, ‘Well, I told her to do hospice and didn’t offer dialysis because of this reason’ and the other nephrologist will be like, ‘Well, why?’ It just seems like—I mean some of it might be also that you’re afraid of what your peers would think of you for doing something like that because I think everyone has a different viewpoint about quality of life and what a patient should or should not endure.” [American nephrologist]^10^  “[conservative management] does take some collaboration between us and primary doctors and other supports…we as a nephrology division can’t do [CM] on our own…without any of those additional services to help out.”^11^ |
| Care setting | “It’s a full spectrum program … That you’re broadly concerned with me as a whole person, a holistic approach … It’s what I wanted to hear.”^6^  “That’s the most positive statement I’ve heard so far … That my health care team will continue to follow me no matter where on the spectrum we go”^6^ |
| **Theme 4: National and international factors** | |
| Limited clinical guidance and training on conservative management | “We really don’t know who’s going to do well and who doesn’t. So I always err on the side of—at least give them a trial, see how it goes” [American nephrologist]^10^  “We have very little, if anything, about patient reported outcome measures, quality of life measures. That’s a big deficiency across the system.”^10^  “We do discuss nondialytic approaches to CKD 5; however I think that there’s huge variability in who really lays that out for them. [Some] don’t seem to either believe in it, or they don’t feel comfortable in approaching that topic and opening up that level of discussion, which is again, you know just a lack of uniformity in the way we approach some … patients.”^11^ |
| Financial incentives to dialyse patients | “I think that unfortunately, dialysis is very financially rewarding to dialysis centers and a lot of caregivers. I feel there are a lot of physicians who just feel that, ‘We’re going to keep people alive as long as possible, and because it’s an option, we’re just going to do it.’” [American nephrologist]^10^  “Right now, the way it’s reimbursed a nephrologist is actually acting against their best financial interest to do what is best for the patient’s interest. It’s never good when you create that kind of misalignment of incentive in the system.” (Fellow)^14^  “There’s no economic incentive [to present a non-dialytic care option]. You have other pressures.” [American nephrologist]^10^ |
| Lack of reliable prognostic tools | “We really don’t know who’s going to do well and who doesn’t. So I always err on the side of – at least give them a trial see how it goes.”^10^  “some patients lived surprisingly longer on dialysis and had a better quality of life than their nephrologists would have predicted”^13^ |

**References**

1. Saeed F, Adams H, Epstein RM. Matters of Life and Death: Why Do Older Patients Choose Conservative Management? *American journal of nephrology*. 2020;51(1):35-42. doi:<https://dx.doi.org/10.1159/000504692>

2. Eneanya ND, Labbe AK, Stallings TL, et al. Caring for older patients with advanced chronic kidney disease and considering their needs: a qualitative study. *BMC nephrology*. 2020;21(1):1-8. doi:10.1186/s12882-020-01870-1

3. Han E, Haldane V, Koh JJK, et al. Perspectives on decision making amongst older people with end-stage renal disease and caregivers in Singapore: A qualitative study. *Health expectations : an international journal of public participation in health care and health policy*. 2019;22(5):1100-1110. doi:<https://dx.doi.org/10.1111/hex.12943>

4. Johnston S, Noble H. Factors influencing patients with stage 5 chronic kidney disease to opt for conservative management: A practitioner research study. *Journal of Clinical Nursing*. 2012;21(9-10):1215-1222. doi:<http://dx.doi.org/10.1111/j.1365-2702.2011.04001.x>

5. Karlin J, Chesla CA, Grubbs V. Dialysis or Death: A Qualitative Study of Older Patients' and Their Families' Understanding of Kidney Failure Treatment Options in a US Public Hospital Setting. *Kidney Med*. 2019;1(3):124-130. doi:<http://dx.doi.org/10.1016/j.xkme.2019.04.003>

6. Oestreich T, Sayre G, O'Hare AM, Curtis JR, Wong SPY. Perspectives on Conservative Care in Advanced Kidney Disease: A Qualitative Study of US Patients and Family Members. *American journal of kidney diseases : the official journal of the National Kidney Foundation*. 2021;77(3):355-364.e1. doi:<https://dx.doi.org/10.1053/j.ajkd.2020.07.026>

7. Seah AST, Tan F, Srinivas S, Wu HY, Griva K. Opting out of dialysis - Exploring patients' decisions to forego dialysis in favour of conservative non-dialytic management for end-stage renal disease. *Health expectations : an international journal of public participation in health care and health policy*. 2015;18(5):1018-29. doi:<https://dx.doi.org/10.1111/hex.12075>

8. Selman LE, Bristowe K, Higginson IJ, Murtagh FEM. The views and experiences of older people with conservatively managed renal failure: A qualitative study of communication, information and decision-making. *BMC nephrology*. 2019;20(1):38. doi:<http://dx.doi.org/10.1186/s12882-019-1230-4>

9. Tonkin-Crine S, Okamoto I, Leydon GM, et al. Understanding by older patients of dialysis and conservative management for chronic kidney failure. *American journal of kidney diseases : the official journal of the National Kidney Foundation*. 2015;65(3):443-50. Comment in: Am J Kidney Dis. 2015 Mar;65(3):372-4; PMID: 25704042 [<https://www.ncbi.nlm.nih.gov/pubmed/25704042>]. doi:<https://dx.doi.org/10.1053/j.ajkd.2014.08.011>

10. Grubbs V, Tuot DS, Powe NR, O'Donoghue D, Chesla CA. System-Level Barriers and Facilitators for Foregoing or Withdrawing Dialysis: A Qualitative Study of Nephrologists in the United States and England. *American Journal of Kidney Diseases*. 2017;70(5):602-610. doi:<http://dx.doi.org/10.1053/j.ajkd.2016.12.015>

11. Ladin K, Pandya R, Kannam A, et al. Discussing Conservative Management With Older Patients With CKD: An Interview Study of Nephrologists. *American Journal of Kidney Diseases*. 2018;71(5):627-635. doi:<http://dx.doi.org/10.1053/j.ajkd.2017.11.011>

12. Noble H, Brazil K, Burns A, et al. Clinician views of patient decisional conflict when deciding between dialysis and conservative management: Qualitative findings from the PAlliative Care in chronic Kidney diSease (PACKS) study. *Palliative Medicine*. 2017;31(10):921-931. doi:<http://dx.doi.org/10.1177/0269216317704625>

13. Wachterman MW, Leveille T, Keating NL, Simon SR, Waikar SS, Bokhour B. Nephrologists' emotional burden regarding decision-making about dialysis initiation in older adults: a qualitative study. *BMC nephrology*. 2019;20(1):385. doi:<https://dx.doi.org/10.1186/s12882-019-1565-x>

14. St Clair Russell J, Oliverio A, Paulus A. Barriers to Conservative Management Conversations: Perceptions of Nephrologists and Fellows-in-Training. *Journal of palliative medicine*. 2021;doi:<http://dx.doi.org/10.1089/jpm.2020.0690>

15. Scott J, Owen-Smith A, Tonkin-Crine S, et al. Decision-making for people with dementia and advanced kidney disease: A secondary qualitative analysis of interviews from the Conservative Kidney Management Assessment of Practice Patterns Study. *BMJ Open*. 2018;8(11):e022385. doi:<http://dx.doi.org/10.1136/bmjopen-2018-022385>
